# Diagnostic capabilities of transperineal ultrasound (TPUS) to evaluate anal sphincter defect post obstetric anal sphincter injury (OASIS)? A systematic review

**DOI:** 10.1007/s40477-022-00763-3

**Published:** 2023-01-11

**Authors:** Surahman Hakim, Budi Iman Santoso, Suskhan Djusad, Fernandi Moegni, Raymond Surya, Andrew Pratama Kurniawan

**Affiliations:** 1 Urogynecology and Pelvic Reconstruction Division, Obstetric and Gynecology Department of Ciptomangunkusumo Hospital, Jakarta, Indonesia; 2 Obstetric and Gynecology Department of Ciptomangunkusumo Hospital, Jakarta, Indonesia; 3 Faculty of Medicine, University of Indonesia, Jakarta, Indonesia

**Keywords:** 3D transperineal ultrasound, 3D endoanal ultrasound, Anal sphincter defect, OASI, Perineal rupture, Ultrasound

## Abstract

**Introduction:**

Endoanal ultrasound (3D-EAUS) is the gold standard imaging investigation for evaluating the anal sphincter; unfortunately, it is not universally available in most obstetric units. This study aims to appraise the ability of transperineal ultrasound (TPUS) compared with 3D-EAUS as the gold standard to identify anal sphincter defects after primary repair of OASIS.

**Methods:**

A systematic search of major databases to identify diagnostic accuracy of 3D-TPUS in evaluating anal sphincter defects. Preferred Reporting Items for Systematic Reviews and Meta-Analyses (PRISMA) guidelines were designed for this systematic review. The risk of bias and applicability concerns were assessed using the QUADAS-2 tool. Our eligibility criteria are patients with a history of primary repair of anal sphincter injuries (OASIS). They were followed up after the primary repair to detect the anal sphincter defect using 3D-TPUS vs. 3D-EAUS as a gold standard.

**Results:**

Two eligible observational studies were included and assessed for risk of bias using the QUADAS-2 tool and showed a low risk of bias and a low risk of concerns. 3D-TPUS had various sensitivity to detect external anal sphincter defects in two studies; meanwhile, the specificity was around 67–70%. For detecting the internal anal sphincter defects, 3D-TPUS had low sensitivity but high specificity (93–94%).

**Conclusion:**

3D-TPUS had various sensitivity to detect external anal sphincter defects and low sensitivity to detect internal anal sphincter defects. On the other hand, 3D-TPUS had low specificity for detecting external anal sphincter defects and high specificity for detecting internal anal sphincter defects.

## Introduction

The incidence of fecal incontinence (FI) after obstetric anal sphincter injury (OASIS) ranges from 9 to 60% [1, 2]. Correct diagnosis and proper suturing of the anal sphincter after delivery are essential to preserve the function [3, 4]. Several societies, such as International Consultation on Incontinence (ICI), International Continence Society (ICS), and International Urogynecological Association (IUGA), stated that endoanal ultrasound (3D-EAUS/EAUS) is the reference gold standard imaging investigation for evaluation the anal sphincter defects [5, 6]. It is commonly used by colorectal surgeons; unfortunately, it is not universally available in most obstetric units, especially urogynecologists. Obstetricians often use perineal and vaginal ultrasound as a more straightforward, cheaper, and reliable method to follow up on the result of suturing the OASIS. Apart from that, vaginal or perineal ultrasound can use endovaginal and abdominal probes, which are more available in obstetrics gynecological units, and the examination is associated with less discomfort [7].

With ultrasound imaging advances, 3- and 4-dimensional technology is also increasingly popular. The advantages include multiplanar imaging, short examination times, and digital volume shortage for later reanalysis [8]. Therefore, this study would like to appraise the ability of 3D-EAUS as the gold standard with 3D transperineal US (3D-TPUS/TPUS) to detect the defect after primary repair of OASIS.

### Review question

How is the accuracy of 3D-TPUS compared with 3D-EAUS after primary repair of OASIS in women diagnosing anal sphincter complex defect?

## Methods

**Fig. 1:**
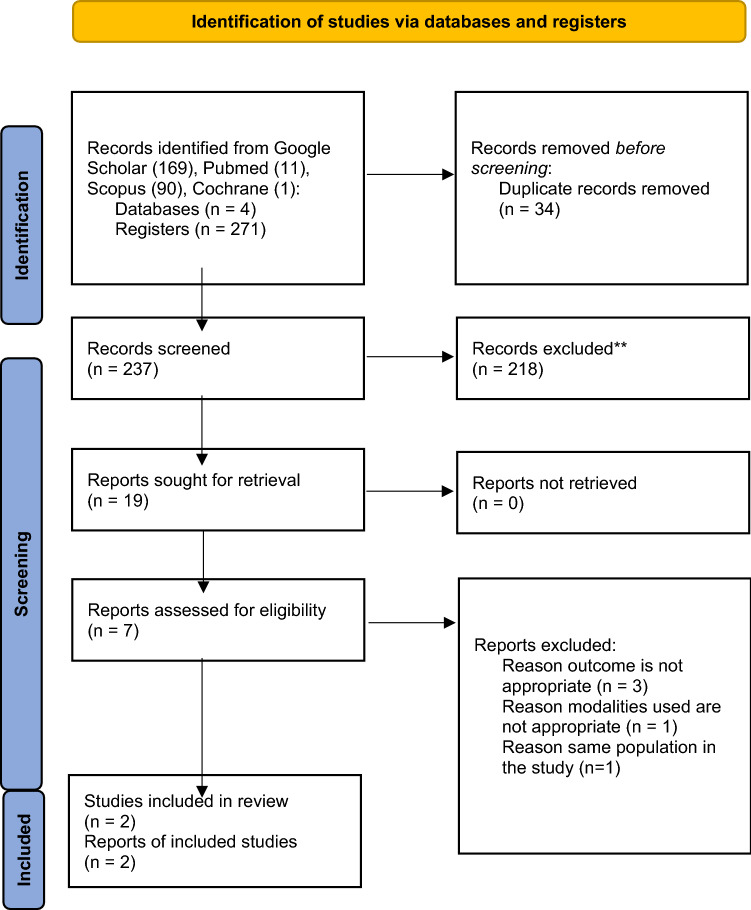
PRISMA Model of literature searching

We used the Preferred Reporting Items for Systematic Reviews and Meta-Analyses (PRISMA) guidelines to design the systematic review. To answer the review question above, eligibility criteria were determined by authors namely detection of 3D-TPUS vs 3D-EAUS as gold standard after primary repair of OASIS and it included the diagnostic studies. Publications before May 2022 were collected. Our search yielded 235 studies across PUBMED, Cochrane, Google Scholar, and Scopus. The inclusion criteria for the studies were 3D-TPUS and 3D-EAUS, women with a history of primary repair of OASIS, and the study evaluating anal sphincter complex with or without FI. We excluded peripartum studies and studies that did not compare between 3D-TPUS and 3D-EAUS. Of the total of 235 studies, only two studies that fulfilled the criteria and analyzed. The details are on the PRISM Flow Diagram Fig. 1.

Ethics approval does not apply for this manuscript since there are no human or animal participant that were used in this systematic review.

The studies were searched and extracted by two investigators (RS) and (APK). We extract sensitivity (Sn), specificity (Sp), positive predictive value (PPV), negative predictive value (NPV), total participant data, and all outcomes measured for 3D-EAUS and 3D-TPUS groups. The study data were further analyzed to 2 × 2 table, and QUADAS-2 tool was used to identify the risk of bias and applicability concerns. All disputes between the two reviewers were discussed and solved by the third reviewer (SH).

Statistical analysis was conducted using Review Manager (RevMan) version 5.1.7 and presented through paired forest plots, one for sensitivity and one for specificity. All values of all true positive (TP), false positive (FP), true negative (TN), and false negative (FN) are listed on the forest plot diagram. The standardized mean of difference with 95% confidence interval (CI) was used to assess the diagnostic test effect. Authors were contacted directly if further information was needed.

## Results

The searching yielded 237 literature from various databases. After the duplication, inclusion, and exclusion screening, only two studies are eligible for further analysis. The five studies were excluded because the outcome was not as expected, the different modalities used, and similar population. The Volloyhaug, et al. study had the same population as Taithongchai et al. Second, the Stuart et al. and Ignell et al. study’s outcome was different from the objective. They were to find the correlation between TPUS, EAUS, anatomy defect, and Waxner score. Martinez et al. correlate the Starck and Nordeval score results from EAUS, TPUS, and Tomographic ultrasound imaging (TUI), but no sensitivity and specificity results. We had sent an email to them requesting more detailed data to be included in our reviews; however, there were no responses. At last, Luo Yijia et al. compared transperineal ultrasound with Magnetic Resonance Imaging (MRI), which is inappropriate for our systematic review. This systematic review thoroughly analyzed only the study from Ros et al. and Taithongchai et al. The literature searching process is detailed in the PRISMA diagram in Fig. 1.

The studies included 305 women after OASIS and were evaluated some months and years after primary repair. Both cross-sectional studies were done in European countries (UK and Spain), with a similar patient initial condition and sampling design. Although Ros et al. study had a very long follow-up duration, they tried to minimize the bias by only including delivered once women, convincing that there was no additional obstetrical trauma during the long follow-up. As in conflict of interest, Ros et al. study openly acknowledged that the BK Medical supported the study by loaning the Ultraview-800 equipment, which is the endoanal ultrasound used. On the other hand, no funding source was declared by Taithongchai et al. The study characteristics and details are in Table 1.

**Table 1:** Characteristics of the study included by the systematic reviews

Author (year)	Participant	Location, time and method of sampling	Inclusion Criteria	Time of followed up after delivery	Instrument specification	Anal sphincter defect definition
Ros et al. [9] (2016)	55 patients with a history of primary sutured of OASIS	Hospital Clinic, de Barcelona, Spain September 2012 to December 2014 Consecutive sampling	All patients with a history of primary repair third or fourth degree of OASIS First delivery	1366 ± 562 days	3D-TPUS -microconvex endocavity probe (type RIC5-9, Voluson-V730 Expert, GE) 3D-EAUS—type 2052, Ultraview-800 BK-Medical at 13 MHz	A discontinuity in the sonographic appearance of the IAS and EAS The extension of the defects were scored by Starck
Taithongchai et al. [7] (2019)	250 women after primary suture of OASIS followed up	Urogynecology center of Croydon University Hospital, United Kingdom October 2013 to August 2015 Consecutive sampling	All women who at least 18 years old Could read and understand english well All women after primary suture of OASIS	The median of 5 months after delivery and repair	3D TPUS used a 3-dimensional 4–8.5 MHz curved array abdominal probe GE Voluson 1 system 3D EAUS used Profocus 2022 of Flex-focus 500 ultrasound system with a 12–16 MHz anorectal transducer	Any defect of ≥ 30 degrees of partial or full thickness in IAS or EAS presenting at ≥ 1 level of slices The severity measured by Starck classification

**Fig. 2:**
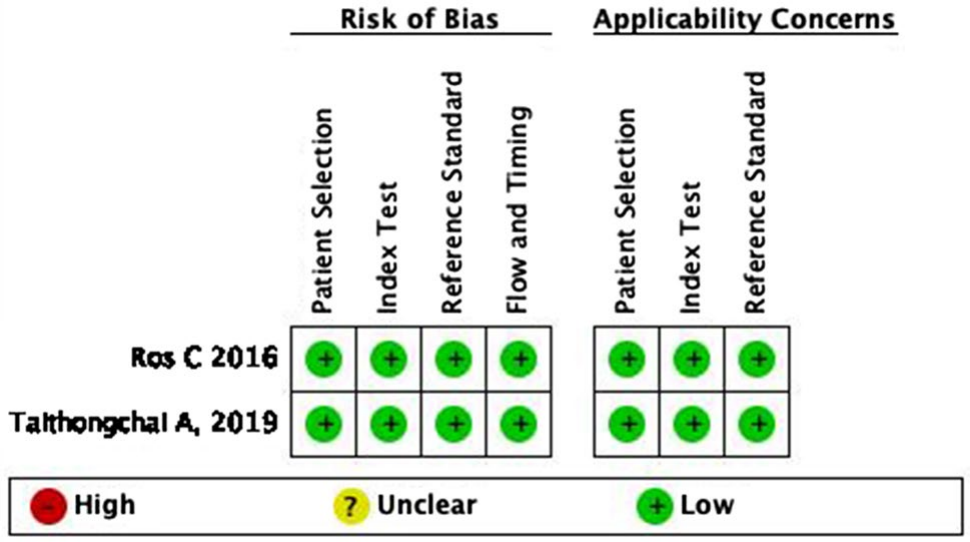
The studies risk of bias assessment using QUADAS-2

Both studies stated a straightforward method of patient selection, the instrument used, and the flow and timing. The risk of bias assessment result is in Fig. 2. Since ultrasound examination is operator-dependent, there is a high risk of result bias in the study. However, both studies minimized the bias by blinding and using multiple investigators. Taithongchai et al. stated that all ultrasound examinations were performed by a single investigator experienced in imaging the anal sphincter. However, three independent investigators were blinded to the clinical and imaging assessment. Every investigator is needed to evaluate the images, and an intraclass correlation analysis was performed to assess agreement. Ros et al. study stated that two investigators did the ultrasound examination, and another two investigators evaluated the images offline for each modality (two for 3D-TPUS and two for 3D-EAUS). In case of discrepancy, two more experts analyzed the volume. All investigators were blinded from the patients' clinical information based on three different specialties (gynecology, radiology, and colorectal surgery) and worked in five different centers.

**Fig. 3:**
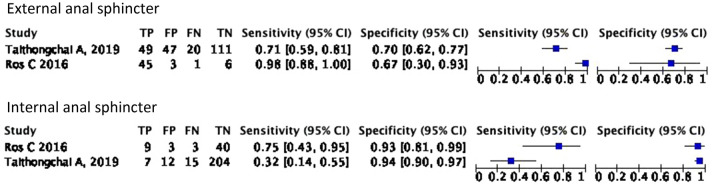
Forest plot of diagnostic capabilities of TPUS in detecting anal sphincter defect for external anal sphincter (EAS) and Internal anal sphincter (IAS)

The sensitivity of TPUS in detecting external anal sphincter defects based on Ros et al. study is 98% higher than Taithongchai et al. study 71%. On the other hand, the specificity to detect a defect on EAS is similar in both studies 67% and 70%, respectively. Furthermore, the sensitivity of TPUS to detect a defect in IAS is pretty low. Ros et al. study found the sensitivity is 75%, and Taithongchai et al. study stated 32% with a large confidence interval. However, the specificity of TPUS to detect a defect on IAS is good, about 93% and 94%. Fig. 3 Summarize the findings into a forest plot table.

## Discussion

OASIS is a nightmare for laboring women. The impact of OASIS could disturb overall women’s quality of life with symptoms such as fecal incontinence, pelvic floor, sexual dysfunction, perineal pain, and rectovaginal fistula [10–12]. A prompt repair during rupture is essential to minimize the risk of complications. However, a follow-up examination focuses more on evaluating the symptoms, sequelae, and decisions regarding future deliveries. RCOG and ACOG recommend that if resources and facilities are available, women with OASIS should undergo endoanal ultrasonography and anal manometry to decide on future deliveries [13, 14]. Unfortunately, endoanal ultrasonography and anal manometry are not convenient for postpartum women and it is usually conducted by colorectal surgeon, not a gynecologist. A woman with the symptom of incontinence, severe anatomical defect on anal sphincter or levator ani avulsion, and low pressure of anal manometry is recommended to have an elective cesarean delivery [13, 15]. Thus, emphasizing the importance of imaging examination on post-repair OASIS women.

Endoanal ultrasound as a gold standard instrument to examine the anal sphincter is very expensive and requires subsequent years of training. Lately, TPUS is widely more available and has started to be routinely used to examine the anal sphincter [15, 16]. The advantages of using TPUS are higher patient applicability with its non-intrusive method, less distortion of the anal canal by the transducer, more affordable, and more straightforward interpretation. TPUS also examines the anal sphincter in relaxed conditions and evaluates the levator ani muscle [17].

The main disadvantages of TPUS are the lack of evidence about the instrument sensitivity and specificity compared to the gold standard. Furthermore, a method to describe the severity and significance of anal sphincter defects by TPUS is not widely accepted and validated yet. One of the most commonly used is Starck classification to assess the anal sphincter defect severity. However, the study that developed the scoring system used endoanal ultrasound [18]. Ros et al. tried to validate the scoring by comparing its means to other modalities and found no significant difference between 3D-TPUS (5.13 ± 3.50) and EAUS (4.87 ± 3.59) although it is a very crude way to compare since Starck scoring consisted of three elements [9]. Another study by Stuart et al. correlate the EAUS Starck scoring result with resting-state TPUS with low to moderate correlation for EAS in length (*r* 0.47 *p* < 0.001), depth (*r* 0.71 *p* < 0.001), and angle (*r* 0.64 *p* < 0.001) [19]. The IAS Starck correlation between EAUS and TPUS for each components were, length (*r* 0.43 *p* = 0.002), depth (*r* 0.56 *p* < 0.001), and angle (*r* 0.56 *p* < 0.001) [19]. The low-moderate correlation was perhaps because of the limitation of the probe shape and limited visualization extent in one image than the EAUS. A new anal sphincter severity scoring and classification is needed for the transperineal ultrasound.

Significant anal sphincter defects are usually described as the EAS/IAS discontinuity with ≥ 30^◦^ angle. Our systematic review founds that the sensitivity and specificity for 3D-TPUS to detect external anal sphincter defect is 71–98% with a specificity of 67–70%. A similar study by Oom et al. evaluated 55 women with FI, compared 3D-TPUS with 2D-EAUS and found that 3D-TPUS has a suitable interobserver Cohen’s Kappa Efficient for EAS (0.63) and IAS (0.78)0.20 However, in detecting IAS the sensitivity is very low 35–72%, but high specificity 93–94%. From our analysis, we concluded that TPUS have good capabilities to screen EAS defect and diagnose for IAS defect based on consistent sensitivity and specificity of 3D TPUS between studies [7, 20].

Some ultrasound has an added feature of tomographic ultrasound imaging (TUI), which gives ultrasound the capabilities to take a multi-slice picture of the anal sphincter. The widely accepted diagnostic criteria of significant external anal sphincter defects using TUI is 30 degrees circumference in the minimum of 2/3 or 4/6 slices and a discontinuity in 1 slice for IAS [21, 22]. Furthermore, Taithongchai et al. (2019) found by including subcutaneous component (the 7th slices) there is a significant improvement of diagnostic accuracy in EAS defect. Therefore a minimum of 3/7 slices of EAS and 2/5 slices of IAS had the best sensitivity and specificity (65% and 75% for EAS, respectively, and 59% and 84% for IAS, respectively) compared to slices without a subcutaneous component in TUI [7]. With the debatable role of subcutaneous tissue on incontinence, Subramaniam et al. stated that no added value is given by including subcutaneous slices to diagnose EAS defects [23, 24]. At last, based on Taithongchai et al. study and Martinez et al. studies TUI examinations do not provide any added value to ordinary 3D-TPUS [7, 22].

The limitation of our study is mainly that we do not account for the women's symptoms because it is hard to evaluate fecal incontinence symptoms objectively. Furthermore, sometimes women with anal sphincter defects have not developed FI, and the degree does not correlate to the severity [25]. Some scoring methods are used to diagnose and assess FI severity, such as St. Mark scoring and Wexner score. However, both scoring is very subjective and heavily evaluate the patient quality of life. Bischoff et al. study stated from 11 fecal incontinence scoring, only the Krickenbeck scoring system was objectively evaluated to diagnose FI [26].

On the other hand, our systematic review strength is that the literature reviewed had no to little risk of bias and reported their conflict of interest, the tool used to extract and asses the risk of bias is appropriate, and a clear study scope and similar study population. This is also the first review to evaluate the use of 3D-TPUS compared with 3D-EAUS.

## Conclusion

The sensitivity of 3D-TPUS to detect external anal sphincter defect in post-repair OASIS Women is 71–98%, and specificity of 67–70%. The sensitivity and specificity of 3D-TPUS to evaluate IAS defect is 35–72% and 93–94%, respectively. Based on this review, we do recommend the use of 3D-TPUS to screen for EAS and diagnose IAS defects and we strongly recommend that women with FI symptoms after OASIS with no TPUS defect to undergo EAUS and manometry examination.

## Data Availability

The authors confirm that the data supporting the findings of this study are available within the article.

## Abbreviations

FI: Fecal Incontinence
EAUS/3D-EAUS: Endoanal ultrasound
TPUS/3D-TPUS: Transperineal ultrasound
EAS: External anal sphincter
IAS: Internal anal sphincter
OASIS: Obstetric anal sphincter injury
ICI: International Consultation on Incontinence
ICS: International Continence Society
IUGA: International Urogynecological Association
PRISMA: Preferred Reporting Items for Systematic Reviews and Meta-Analyses
SN: Sensitivity
SP: Specificity
PPV: Positive predictive value
NPV: Negative predictive value
FP: False positive
FN: False negative
TUI: Tomographic ultrasound imaging
MRI: Magnetic resonance imaging
